# Comparison of quality of life in patients who underwent mechanical mitral valve replacement: star GK vs SJM

**DOI:** 10.1186/s13019-020-1045-1

**Published:** 2020-01-07

**Authors:** Jiang-Shan Huang, Ning Xu, Kai-Peng Sun, Zhi-Nuan Hong, Liang-Wan Chen, Yur-Ren Kuo, Qiang Chen

**Affiliations:** 1 Department of Cardiovascular Surgery, Union Hospital, Fujian Medical University, Fuzhou, 350001 People’s Republic of China; 2 Department of Cardiac Surgery, Fujian Provincial Maternity and Children’s Hospital, Affiliated Hospital of Fujian Medical University, Fuzhou, 350001 People’s Republic of China; 3 Division of Plastic Surgery, Department of Surgery, Kaohsiung Medical University Hospital, 100 TzYou 1st Rd, Kaohsiung City, 80756 Taiwan

**Keywords:** Mitral valve replacement, Quality of life, Valve noise

## Abstract

**Background:**

We want to compare the impact on health-related quality of life (HRQoL) between the Star GK and the SJM valve in the Chinese population.

**Methods:**

We retrospectively enrolled a total of 172 patients who had undergone mechanical mitral valve replacement (MVR) (SJM valve in 87 patients and Star GK valve in 85 patients) at our institution from January 2013 to December 2015. We measured the sound pressure level, and used 2 self-administered questionnaires and the Chinese version of SF-36 to measure the HRQoL and valve-specific questions to evaluate patient anxiety.

**Results:**

The Star GK group and the SJM group were similar in age, gender, body surface area, diameter of the implanted valve, underlying disease and current median NYHA class. Regarding the valve sound pressure perceived 1 year after operation, the SJM valve was slightly quieter than the Star GK valve, but the sound pressures of the two valves showed no significant differences. No significant differences in any of the eight subscales of the SF-36 were found between the two groups.

**Conclusions:**

The present study suggests that the Star GK valve is similar to the SJM valve in its impact on HRQoL and audibility of mechanical sound in the Chinese population.

## Introduction

In recent years, there have been an increasing number of prosthetic mechanical heart valves implanted for heart valve diseases. The relevant surgical outcome and prosthetic heart valve improvement provide better hemodynamics and durability [1, 2]. After performing document retrieval, we found that few comparative studies on differences in mechanical mitral valve replacement (MVR) materials regarding HRQoL have been conducted, especially in the Chinese population. Thus, we enrolled a total of 172 patients who had undergone MVR using the Star GK mechanical valve or SJM mechanical valve at our institution to compare the HRQoL of these patients.

## Materials and methods

### Study design

Total 172 patients who had undergone mechanical MVR (87 patients with SJM valve and 85 patients with Star GK valve) at our institution from January 2013 to December 2015 were enrolled, and the patients returned to the outpatient department for follow-up visits. The inclusion criteria included the following: (1) first-time MVR using the Star GK valve or SJM valve; (2) no hearing disorders; (3) medical compliance to complete at least a full 1-year follow-up. The exclusion criteria included the following: (1) refusal to undergo routine examination; (2) inability to finish the questionnaires; (3) recipient of other valve replacement procedures or having poor cardiac function. All participants were requested to complete the relevant questionnaires in the outpatient department.

The standard median sternotomy approach was used for all of the patients. MVR was performed under cardiopulmonary bypass (CPB) with cardiac arrest and mild systemic hypothermia (30–32 °C). Follow-up assessments were conducted at the 1st, 3rd, and 12th months of the first year. The assessments included clinical examination, ECG, chest X-ray, and transthoracic echocardiography (TTE). We used a clinical database to obtain the follow-up information and assess postoperative complications, including endocarditis, thromboembolism, bleeding events, valve thrombosis, and reoperation. We defined severe events according to Akins’ report [3]. The target international normalized ratio (INR) for patients with a mechanical mitral valve was between 2 and 3 [4].

### Questionnaires

All participants finished three questionnaires, including the short-form health survey (SF-36), the cardiac anxiety questionnaire (CAQ) and valve-specific questions. We used the Chinese version of the short-form health survey (SF-36) to assess physical and mental quality of life. The SF-36 has already been proven to be a reliable and valid tool in precision studies, and it is widely used in China. This questionnaire consists of 36 items and 8 scales (physical role, physical functioning, vitality, bodily pain, emotional role, social functioning, mental health, and general health) [5–7].

The second questionnaire included valve-specific questions used in the Boston Area Anticoagulation Trial. It includes the following 6 questions: Q1: Taking anticoagulation restricts my life; Q2: I worry a lot about the side effects; Q3: My health is better since taking anticoagulation; Q4: Taking anticoagulation will make my health better in the future; Q5: I feel disturbed by the daily need of anticoagulation; Q6: Are you disturbed by the “clicking” sound of the valve [8]. To fully evaluate the clicking noise made by the mechanical valve, we further asked patients the following questions: Q1: Whether informed about the potential noise made by prosthesis before operation; Q2: When did the noise stop bothering you; Q3: Did you get support when disturbed by the noise [9].

We used a sound level meter (TM, Teichman) to measure the sound level of the mechanical prosthesis. This examination was done in a completely quiet room in the outpatient department after the participant finished all of the questionnaires. Patients were in a supine position, and they were well-rested. The machine was positioned 1 cm from the chest at the lower presternal region where the sound was loudest. We used dB as the unit of sound in this study [10].

### Mechanical valve

In our institution, we used the Star GK mechanical valve and the SJM mechanical valve in MVR procedures. The Star CK valve is double-leaf valve with a bicuspid valve open angle of 85° (China-made valve, Beijing Sida Mechanical Valve Co., Ltd.). Three specifications, 25 mm, 27 mm, and 29 mm, were available, and we chose the specification based on the body surface area (BSA) of the patient.

### Statistical analysis

Continuous variables are expressed as the x ± s. Student’s t-test or analysis of variance was applied for continuous variables, and the χ2 test or Fisher’s test was applied for categorical variables. The scores of the SF-36 were analyzed by t-tests, and the answers to the self-questionnaire were analyzed by chi-squared tests. We defined statistical significance as a *p* value < 0.05.

## Results

The patient characteristics and the valve noise results are shown in Table 1. There were no statistically significant differences in age, sex, body surface area and diameter of the implanted valve between the two groups. The current median New York Heart Association (NYHA) grade was grade II for both groups. The sound of the Star GK prosthetic valve (69.2 ± 6.7) was slightly louder than that of the SJM valve (68.4 ± 6.3). However, there was no significant difference found in sound level between the two groups.

**Table 1:** Demographic, echocardiography variables compared between SJM group and GK group

Item	SJM group (*n* = 87)	GK group (*n* = 85)	*P* value
Age (years)	47.32 ± 9.20	46.95 ± 8.93	0.78
Female	46(52.87%)	43(50.59%)	0.87
Underlying disease			
Mitral stenosis	31(35.63%)	36(42.35%)	0.37
Mitral incompetence	56(64.37%)	49((57.65%)	
Body surface area (cm^2^)	1.84 ± 0.14	1.86 ± 0.13	0.50
Diameter of implanted valve (mm)	27.67 ± 1.45	27.63 ± 1.68	0.18
Current Mitral valve gradient (mmHg)			
25 mm type	15.21 ± 5.41	15.48 ± 5.59	0.92
27 mm type	12.38 ± 5.23	12.41 ± 5.14	0.92
29 mm type	10.34 ± 4.36	10.26 ± 4.28	0.90
Current NYHA (median)	II	II	
Maximum Valve noise (dB)	68.4 ± 6.3	69.2 ± 6.7	0.57

The relevant postoperative complications are shown in Table 2. Endocarditis, thromboembolism, and bleeding events were similar in the two groups. Valve thrombosis and reoperation during the follow-up period did not occur in any of the patients. As shown in Table 3, there was no significant difference found in the scores of the eight subscales of the SF-36 between the Star GK group and SJM group. The results of the valve-specific questions are shown in Table 4. The percentage of suffering from taking anticoagulation and the occurrence of complications, such as bleeding events and embolism, were similar in both groups. Most patients knew the importance of anticoagulation and had an INR between 2.0 and 3.0.

**Table 2:** Postoperative valve-specific complication compared between SJM group and GK group

	SJM group	GK group	*P*-value
Thromboembolism	2/87	3/85	0.63
Bleeding event	4/87	3/85	0.72
Endocarditis	0	1/85	0.31
Valve thrombosis	0	0	NS
Reoperation	0	0	NS

**Table 3:** SF-36 results compared between SJM group and GK group in 1 year after operation

Item	SJM group	GK group	*P* value
Physical functioning	77.09 ± 25.68	75.76 ± 24.05	0.55
Role physical	70.78 ± 20.15	67.76 ± 20.33	0.93
Bodily pain	81.74 ± 24.94	77.36 ± 25.61	0.81
General health	66.20 ± 19.21	63.47 ± 18.94	0.90
Vitality	60.56 ± 20.21	62.91 ± 19.18	0.63
Social functioning	88.14 ± 24.24	87.98 ± 23.20	0.69
Role emotional	77.62 ± 17.14	76.15 ± 16.89	0.90
Mental health	83.45 ± 23.76	79.51 ± 22.45	0.27

**Table 4:** Valve-specific questions compared SJM group and GK group

Question	SJM group		GK group		*P* value
	Never/seldom	Sometimes/often/always	Never/seldom	Sometimes/often/always	
Q1	61/87	26/87	55/85	30/85	0.45
Q2	47/85	40/87	48/85	37/85	0.75
Q3	57/87	30/87	55/85	30/85	0.91
Q4	21/87	66/87	19/85	66/85	0.78
Q5	53/87	34/87	55/85	30/85	0.61
Q6	64/87	23/87	62/85	23/85	0.93

A total of only 19% patients were informed of the mechanical valve sound, and no difference was found between the Star GK group and SJM group. However, at 1 year after operation, there were still 27% patients in the Star GK group who stated that they were sometimes or often disturbed by the clicking sound, and this was similar with the percentage of those in the SJM group who experienced this, which was 26%. Furthermore, only 15% patients in the SJM group and 12% patients in the Star GK group were able to receive enough support when they were disturbed by the “clicking” sound (Table 5).

**Table 5:** Further 3 valve-specific questions compared between SJM group and GK group

Question	Answer	Valve type		*P* value
		SJM	GK	
Q1:whether informed the potential noise made by prosthesis before operation;	yes	16/87	16/85	0.94
	no	71/87	69/85	
Q2:when did the noise stop bothering you	immediately after operation	33/87	28/85	0.88
	by time of discharge after operation	4/87	6/85	
	by 3 months after operation	6/87	8/85	
	by 6 months after operation	10/87	7/85	
	by 9 months after operation	11/87	13/85	
	still bothering	23/87	23/85	
Q3:whether get support from doctor when disturbed by the noise	get enough support	8/54	7/57	0.79
	get little support	29/54	32/57	
	no support	17/54	18/57	

## Discussion

Mechanical valve replacement has already been proven to be a reasonably safe and effective treatment for heart valve diseases. Although the mortality, morbidity, and recurrence rates of such diseases associated with mechanical valve replacement have been analyzed in precision studies, the effect of mechanical valves on the HRQoL has been rarely studied, especially in the Chinese population [1, 2]. HRQoL may be affected by the following factors: the clicking sound of the mechanical valve, the mental state of the patient, and the recognition of anticoagulation-related bleeding events and valve embolism by the patient. Both Star GK valve and SJM valve are bileaflet valve. Hemodynamics, antithrombogenicity, and durability of the Star GK valve and SJM valve have been previously proven to be reliable. Thus, the effect of the valve on the HRQoL and the price should be taken into consideration in the evaluation and selection of a mechanical valve. In this study, we aimed to compare the effect of the Star GK valve and that of the SJM valve on the HRQoL of patients who had undergone MVR.

We hypothesized that the Star GK valve and SJM valve had similar impacts on the HRQoL of patients who had undergone MVR. We used the Chinese version of the SF-36 and found no significant difference between the Star GK valve group and SJM valve group. Although the SF-36 is widely used across disease areas, disease-specific questionnaires should be added to provide more evidence [9].

The valve-specific questions used in the Boston Area Anticoagulation Trial focuses on anticoagulation-related problems. The occurrence rate of bleeding events and embolism was low, and it was similar between the Star GK valve group and SJM valve group. We contribute this finding to the antithrombogenicity and durability of the Star GK valve and SJM valve and to the recognition of anticoagulation by the patients. Most patients knew the importance of anticoagulation, and they took warfarin daily and monitored the INR regularly. At our institution, we adjust the warfarin dose before discharge and during the follow-up period to achieve an INR between 1.7 and 2.3. In the first and second months, we monitored the INR each week. In third and fourth months, we monitored the INR twice per month. In fifth and sixth months, we monitored the INR every 3 weeks. After 6 months after operation, patients needed to monitor the INR only once per month. For this reason, we found most patients were not bothered by taking oral anticoagulation. However, the daily intake and periodic monitoring of the INR made patients constantly aware of anticoagulation-related complications, and this constant monitoring annoyed a large portion of patients in both groups.

The mechanical valve “clicking” sound is another factor that may influence the quality of life of patients. The mechanical valve generates a clicking sound, which is often audible to patients and patients’ relatives, and it may become more clear at night or in quiet rooms [10]. More than half of patients can hear the “clicking” sound made by their mechanical prosthesis [11]. Precision reports described the clicking sound of mechanical valve as a source of disturbance; as a result, some patients experience annoyance, sleeping disorders, concentration disturbance and social embarrassment [2, 10–12]. Faraz Kerendi and colleagues reported that a 55-year-old male even underwent a second replacement due to intolerance of the clicking noise. The patient experienced severe difficulty with the “clicking” noise made by the mechanical valve. Finally, he underwent a second MVR with a bioprosthesis [13]. This report suggested that cardiac surgeons may underestimate the impact of valve noises on the HRQoL of patients, unlike the life-threatening complications related to anticoagulation and thromboembolic events. Therefore, the importance of considering the potential of the clicking sound on the quality of life of patients should be emphasized when choosing the optimal prosthetic valve. Nishi K and colleagues compared the impacts of the ATS valve, SJM valve and CM valve on HRQoL and emphasized that long-lasting valve sounds significantly affect the quality of life of patients. Patients bothered by the clicking sound scored significantly lower scores on the role-physical, bodily pain, vitality, social functioning, role-emotional, and mental health subscales than those who were not bothered by the clicking sound [14]. Thus, it is necessary to choose a quieter mechanical valve.

One study proved that different mechanical valves were different regarding their audibility and showed that different valves produce different sound levels in vitro [15]. Sezai A reported in their study that 84.4% of patients with ATS valves were not aware of valve sounds. ATS valves scored significantly lower than SJM valves on audibility measurements of the valve sound, disturbance during daytime, sleep disturbance, requests for replacement with a soundless prosthetic valve, audibility to others, and noise index. Therefore, they concluded the ATS valve is presently considered to have a better impact on quality of life than other mechanical valves [16]. Pedersen TA and colleagues measured valve sounds from 15 patients with ATS valves, 29 with Medtronic-Hall valves, and 40 with St. Jude mechanical (SJM) valves. They found the highest sound levels were in the ATS valve group and the lowest sound levels were in the SJM valve group. Furthermore, there was larger variation in sound pressure levels in the ATS valve group [17].

In this study, we measured the sound pressure levels from the Star GK valve and SJM valve in the mitral position. The sound pressure level of the Star GK valve was similar to that of the SJM valve. At 1 year after operation, 27% patients with the Star GK valve stated they were sometimes or often bothered by the clicking sound, while the corresponding percentage in the SJM valve group was 26%. The median time for the sound to stop bothering patients was 3 months after operation in both the Star GK valve group and SJM group. This result suggests that the Star GK valve and SJM valve were similarly audible to patients who underwent MVR. However, this is a retrospective investigation, and response options immediately after operation were limited and not as accurate as those at different times of discharge after operation. Thus, it may have resulted in us not finding a difference in the length of time in which the sound stopped bothering patients.

When patients were asked when the clicking sound stopped bothering them, 81% patients in the Star GK valve group and 82% in the SJM valve group complained they were not informed of this issue, and only 13% patients of the total patients could get enough support when disturbed by the persistent noise. Limb D and his colleagues reported that few patients had received any information about the “clicking” noise; thus, when this problem occurred, patients were poorly prepared. They emphasized the importance of informing patients who were prepared to undergo valve replacement about the potential valve noise. It would be more useful to arrange patients to meet someone who had already undergone mechanical valve replacement and communicate the influence of the valve noise before performing the valve replacement [18].

When choosing an optimal mechanical valve, surgeons may need to consider the potential noise disturbances caused by mechanical valves. According to a previous report, female patients younger than 60 years are more likely to be disturbed by the clicking sound than other patients. Careful patient teaching, hearing examinations and stimulation of the valve noise should be integrated into routine preparation for mechanical valve replacement, especially for those younger than 60 years old and for female patients [11, 19].

From the data summarized above, the Star GK valve and SJM valve have a similar impact on HRQoL, and they have a similar audibility of mechanical sounds in the Chinese population. Price should also be taken into consideration when choosing a mechanical valve, especially in developing countries. Rheumatic heart disease is still the most common type of valvular heart disease in China. The difference in price between the Star GK mechanical valve and SJM mechanical valve (8000 vs. 12,900 RMB) may still be large enough to be problematic for some patients in China. Thus, the Star GK mechanical valve is an alternative valve in China for no significant difference in the relevant postoperative complications, audibility and HRQoL.

There are some shortcomings of this study. First, it is retrospective study, not a prospective randomized trial, and it is limited by its retrospective nature and possible deviation in case selection. We still consider our paper also has some clinical significance. Second, this study was performed at a single institution in China, and multicenter cooperation is needed in future studies. Third, the follow-up was only conducted at 1 year after operation, and more detailed and longer follow-up protocols are needed in future studies.

## Conclusion

The present study suggests that the Star GK valve is similar to the SJM valve in regard to the impact on HRQoL and audibility of mechanical sound in the Chinese population. We suggest choosing the one economically more convenient in your center for no significant difference in clinical outcome and audibility. Further studies including a larger sample are necessary to evaluate the HRQoL of these two mechanical valves.

## Data Availability

Data sharing not applicable to this article as no data sets were generated or analyzed during the current study.

## Abbreviations

BMI: Body mass index
BSA: Body surface area
CAQ: Ardiac anxiety questionnaire
CPB: Cardiopulmonary bypass
HRQoL: Health-related quality of life
INR: International normalized ratio
MVR: Mitral valve replacement
SF-36: The short-form health survey
TTE: Transthoracic echocardiography
